# Supplementary material for: Saccharomyces boulardii (CNCM I-745) improves intestinal damage in sepsis by remodeling intestinal flora structure
Source: Front Cell Infect Microbiol. 2025 Jul 31;15:1602792. doi: 10.3389/fcimb.2025.1602792 (PMC12350329; doi:10.3389/fcimb.2025.1602792)
Supplement: Supplementary file 1 [file DataSheet1.zip › summary/summary/8_Advanced_analyse/2_PICRUSt2_result/COG/Group.AW4_vs_AW0/PICRUSt2_COG_Group.AW4_vs_AW0_diff.pdf]

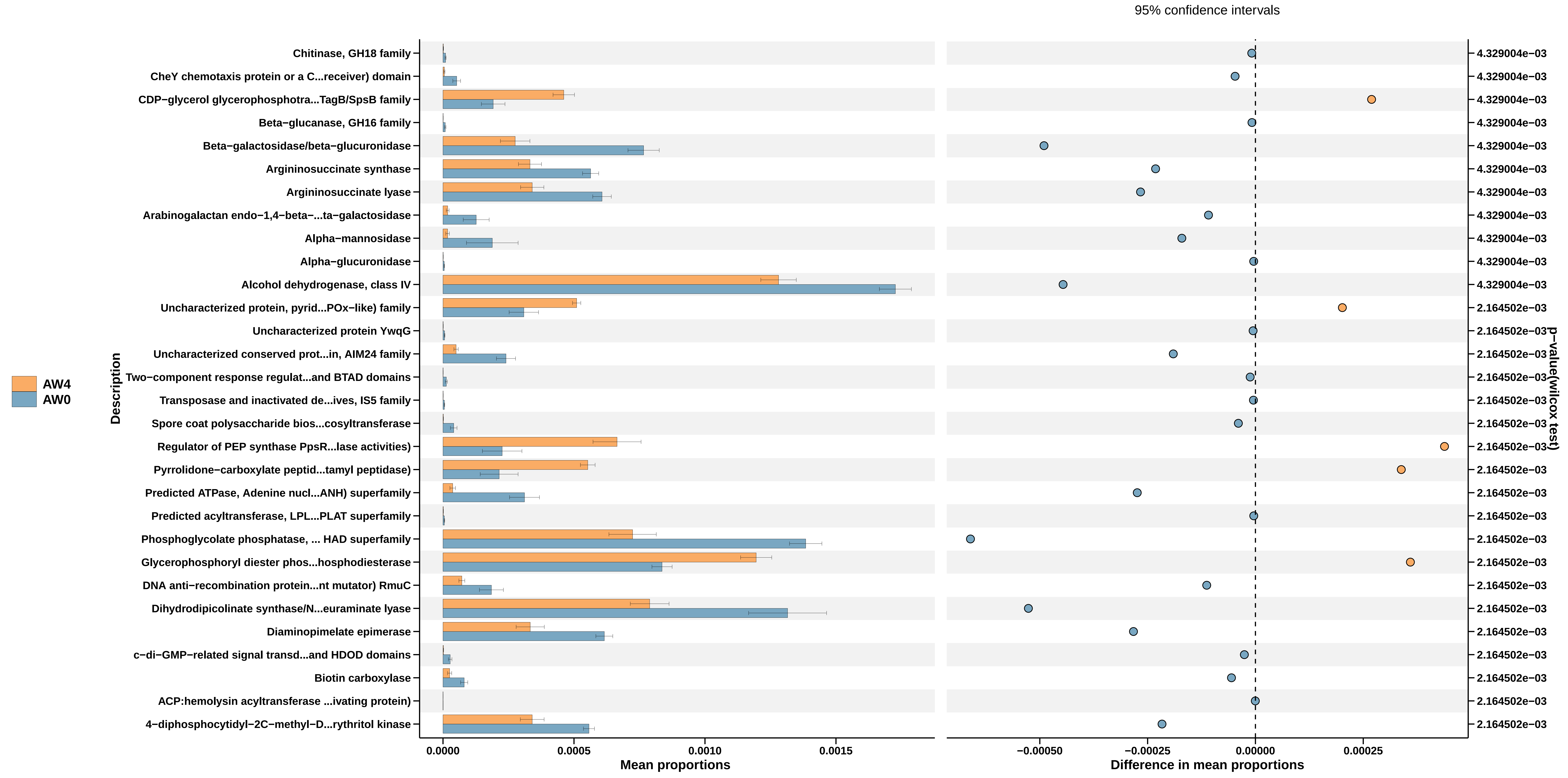
95% confidence intervals
Chitinase, GH18 family
CheY chemotaxis protein or a C...receiver) domain
CDP−glycerol glycerophosphotra...TagB/SpsB family
Beta−glucanase, GH16 family
Beta−galactosidase/beta−glucuronidase
Argininosuccinate synthase
Argininosuccinate lyase
Arabinogalactan endo−1,4−beta−...ta−galactosidase
Alpha−mannosidase
Alpha−glucuronidase
Alcohol dehydrogenase, class IV
Uncharacterized protein, pyrid...POx−like) family
Uncharacterized protein YwqG
Uncharacterized conserved prot...in, AIM24 family
Two−component response regulat...and BTAD domains
Transposase and inactivated de...ives, IS5 family
Spore coat polysaccharide bios...cosyltransferase
Regulator of PEP synthase PpsR...lase activities)
Pyrrolidone−carboxylate peptid...tamyl peptidase)
Predicted ATPase, Adenine nucl...ANH) superfamily
Predicted acyltransferase, LPL...PLAT superfamily
Phosphoglycolate phosphatase, ... HAD superfamily
Glycerophosphoryl diester phos...hosphodiesterase
DNA anti−recombination protein...nt mutator) RmuC
Dihydrodipicolinate synthase/N...euraminate lyase
Diaminopimelate epimerase
c−di−GMP−related signal transd...and HDOD domains
Biotin carboxylase
ACP:hemolysin acyltransferase ...ivating protein)
4−diphosphocytidyl−2C−methyl−D...rythritol kinase
Description
AW4
AW0
0.0000
0.0005
0.0010
0.0015
Mean proportions
−0.00050
−0.00025
0.00000
0.00025
Difference in mean proportions
p−value(wilcox test)
4.329004e−03
4.329004e−03
4.329004e−03
4.329004e−03
4.329004e−03
4.329004e−03
4.329004e−03
4.329004e−03
4.329004e−03
4.329004e−03
4.329004e−03
2.164502e−03
2.164502e−03
2.164502e−03
2.164502e−03
2.164502e−03
2.164502e−03
2.164502e−03
2.164502e−03
2.164502e−03
2.164502e−03
2.164502e−03
2.164502e−03
2.164502e−03
2.164502e−03
2.164502e−03
2.164502e−03
2.164502e−03
2.164502e−03
2.164502e−03
